# Nursing students’ readiness and training needs for interprofessional education: A cross-sectional study in South Korea

**DOI:** 10.1371/journal.pone.0342320

**Published:** 2026-04-21

**Authors:** Sun-Yi Yang

**Affiliations:** Konyang University College of Nursing, Konyang University, Daejeon, Republic of Korea; Alexandria University Faculty of Nursing, EGYPT

## Abstract

The significance of interprofessional education (IPE) in fostering interprofessional collaboration is being increasingly emphasized in healthcare. This study analyzed the training needs of nursing students based on the Interprofessional Education Collaborative Core Competencies using a descriptive, cross-sectional design. A structured survey of 108 fourth-year nursing students in Korea was conducted between June and July 2024. Data were analyzed with SPSS 27.0, using the Borich needs assessment model and importance-performance analysis (IPA). The analysis identified a critical need for focused educational intervention to enhance collaborative skills. Specifically, the results highlighted Teams and Teamwork and Roles and Responsibilities as priority areas for improvement, with a need for students to better define and differentiate roles, communicate with clarity and cultural humility, and advance team reasoning and shared leadership strategies. The findings indicate that the Core Competencies are not sufficiently integrated into nursing curricula to meet nursing students’ training needs. Therefore, curriculum developers must prioritize focused programs that enhance nursing students’ understanding of their roles, responsibilities, and cooperative strategies to effectively integrate IPE into nursing education and bridge these professional gaps. Convenience sampling and reliance on self-report data are acknowledged as limitations of this study.

## Introduction

As modern healthcare has become highly specialized and segmented, the provision of efficient and safe patient-centered healthcare services has become possible, but only when healthcare workers form a cohesive and cooperative team [1]. However, this increasing specialization can lead to a fragmentation of care, where professionals may lack a comprehensive understanding of the patient’s overall needs and treatment plan [2]. This dearth of a unified approach, often stemming from an absence of clear understanding of professional roles and responsibilities, can result in communication breakdowns, medical errors, and lower patient satisfaction [1].

Interprofessional team-based care is streamlined, improves patient health, and enhances values throughout healthcare systems overall, as it hinges on effective teamwork [3]. The importance of interprofessional education (IPE) is being increasingly emphasized in the healthcare sector to achieve interprofessional collaboration (IPC) [4]. However, it is important to note that IPE is not a monolithic concept, and the various forms it takes—such as simulation-based training, clinical-setting-based learning, and online modules—may yield different learning outcomes. Understanding these different approaches is essential for effective curriculum development [2,4]. According to the World Health Organization (WHO) [4], IPC in education and practice can play a vital role in mitigating the global health workforce crisis; moreover, the WHO is focusing on expanding IPE as part of an effort to meet the needs of future healthcare systems [2].

IPE is a process in which learners specializing in two or more disciplines learn about one another’s respective fields, as well as about their unique roles and responsibilities, to improve patient health [5]. Through this process, learners can cultivate interprofessional communication skills and develop the essential teamwork competencies needed to provide precise and safe healthcare services, ultimately helping to improve patient safety and health [6].

### Background

Interprofessional education (IPE) is widely recognized as a means of fostering collaborative healthcare, which is crucial for effective patient care [7]. A systematic literature review affirmed that IPE positively changes attitudes toward team-based healthcare and promotes collaborative behaviors [8]. Crucially, IPE is linked to improved clinical outcomes, such as reduced adverse drug reactions, lower emergency room visits and readmission rates, improved treatment outcomes, and enhanced patient satisfaction [9]. These findings underscore that proactive collaboration among healthcare professions, rather than professional segmentation, is essential for patient safety and care quality [5].

To operationalize this, the Interprofessional Education Collaborative (IPEC) in the US [10] and similar bodies in Europe, Australia, and Canada have actively integrated IPE into undergraduate curricula [7]. The IPEC, which guides collaborative patient care, specifies four Core Competencies (CCs): Values and Ethics, Roles and Responsibilities, Communication, and Teams and Teamwork [2] (hereafter “interprofessional CCs”).

To develop effective IPE simulation programs, it is first necessary to systematically assess healthcare students’ readiness for IPE and their perceived importance of and performance in the interprofessional CCs. Whereas countries such as the US and Japan have made various efforts to develop and validate IPE programs [8,11] and integrate IPE into accreditation standards [12], South Korea still faces significant gaps. Studies have been conducted on the perceptions of faculty [13,14] and pharmacy students [5] regarding IPE readiness and interprofessional CCs, but data on the training needs and perceptions of nursing students—the largest healthcare workforce—remain insufficiently comprehensive. Current IPE programs in South Korea, while growing in number [15,16], often lack a systematic framework based on a robust needs assessment, which is essential for developing effective and evidence-based curricula.

To precisely determine the necessary educational focus, this study employed importance-performance analysis (IPA), which is valuable for practical priority setting [17], alongside the Borich needs assessment model. While both methods have been used separately in nursing education research and program design [18–20], this study enhances rigor and practical utility by combining them to analyze IPE training needs. Specifically, IPA maps the gap between students’ perceived importance (expected state) of and current performance (current state) in interprofessional CCs onto a 4-quadrant Locus for Focus Model (LfFM) [21]. The Borich needs assessment model then provides a consolidated score for training necessity. This approach will identify the interprofessional CCs in which nursing students show low performance despite perceiving high importance, thereby accurately quantifying the training demand and deriving robust, data-driven priorities for developing IPE simulation programs in healthcare.

This study was conducted to identify nursing students’ training needs to develop IPE simulation programs in healthcare. As such, this study aimed to identify the following:

- Nursing students’ perceptions of and readiness for IPE, in addition to their views on the importance of and their performance in interprofessional CCs.
- The gap between nursing students’ perceived importance of and performance in interprofessional CCs according to their general characteristics.
- The gap between nursing students’ perceived importance of and performance in interprofessional CCs using IPA.
- Nursing students’ priorities in terms of their IPE training needs using the LfFM and the Borich needs assessment.

## Materials and methods

### Research design

This research utilized a descriptive, cross-sectional design to identify nursing students’ perceptions of and readiness for IPE. It also explored the training topics that learners need to cover according to interprofessional CCs, aiming to develop an IPE simulation program for healthcare. It should be noted that this study assessed the needs for IPE simulation programs; it does not report on delivering a simulation program.

### Research participants

The target population consisted of fourth-year undergraduate nursing students in South Korea. This study used a convenience sampling method to recruit participants. Inclusion criteria were students who had completed at least two semesters of clinical practice courses. Exclusion criteria included students with a minimum of one year of prior professional experience in healthcare before entering the nursing program (as such experience could influence the results) and those from the primary investigator’s university (to ensure research autonomy). Participants were recruited via an online survey link distributed on various online student platforms, and all participants provided online informed consent before starting the survey.

The sample size was determined using G*power 3.1.9 software [22], which indicated a minimum sample size of 90 participants based on a paired *t*-test with an effect size of 0.3, a significance level of  .05, and a power of 0.8. Considering attrition rates of 3.6 − 20% reported in previous studies [7,23] a total of 108 participants were selected through convenience sampling for the final analysis.

### Research tools

#### Perceptions of IPE.

Nursing students’ perceptions of IPE were measured using the Interdisciplinary Education Perception Scale developed by Luecht et al. [24] and revised by McFadyen et al. [25].

The Interdisciplinary Education Perception Scale comprises 18 items divided into four sub-domains: perception of competency and autonomy (8 items), perceived need for cooperation (2 items), perception of actual cooperation (5 items), and understanding of others’ values (3 items). Each item is rated on a 6-point Likert scale, ranging from *strongly disagree* (1 point) to *strongly agree* (6 points). The total score ranged from 18 to 108 points, with higher scores indicating more positive perceptions of IPE. Cronbach’s α for reliability was  .92 in this study.

#### Readiness for IPE.

Readiness for IPE was assessed using the Readiness for Interprofessional Learning Scale developed by Parsell and Bligh [26], modified by McFadyen et al. [27], and translated into Korean by Kim and Kim [28].

The Readiness for Interprofessional Learning Scale includes 19 items across 4 domains: teamwork and collaboration (9 items), negative professional identity (3 items), positive professional identity (4 items), and Roles and Responsibilities (3 items). Each item is rated on a 5-point Likert scale, from *strongly disagree* (1 point) to *strongly agree* (5 points). The total score ranges from 19 to 95 points, with higher scores indicating a greater readiness for IPE. Cronbach’s α was  .85 in the current study.

#### Perceptions of the importance of interprofessional CCs.

This study included 33 sub-competency statements across 4 domains to assess nursing students’ perceptions of the importance of interprofessional CCs: Values and Ethics (11 items), Roles and Responsibilities (5 items), Communication (7 items), and Teams and Teamwork (10 items). Each item was evaluated on a 5-point Likert scale, from *very low* (1 point) to *very high* (5 points). The total score ranged from 33 to 165 points, with higher scores indicating greater perceived importance of the CCs. Reliability verification during tool development (established through an agreement between the nurses and nursing faculty) demonstrated a correlation coefficient of r = .72–.84, and Cronbach’s α was  .98 in this study.

#### Performance in interprofessional CCs.

This study evaluated nursing students’ performance in interprofessional CCs using 33 sub-competency statements distributed across four domains, as presented by the IPEC [10]: Values and Ethics (11 items); Roles and Responsibilities (5 items); Communication (7 items); and Teams and Teamwork (10 items). Each item was rated on a 5-point Likert scale, ranging from *very low* (1 point) to *very high* (5 points). Total scores ranged from 33 to 165 points, with higher scores indicating better performance in interprofessional CCs. Cronbach’s α in this study was  .98.

#### Analysis of priorities for training needs to enhance interprofessional CCs.

This study applied both the Borich needs assessment model and the LfFM to categorize CCs into four quadrants, based on performance and the discrepancy between perceived importance and actual performance. The quadrants were defined as follows:

Quadrant A (Concentrate here): items with above-average importance and discrepancy.

Quadrant B (Keep up the good work): items with average importance but below-average discrepancy.

Quadrant C (Low priority): items with below-average importance and below-average discrepancy.

Quadrant D (Possible overkill): items with below-average importance but above-average discrepancy.

### Data collection

The study received Institutional Review Board (IRB) approval from Konyang University (IRB #KYU 2024-05-003; date: June, 12, 2024). The IRB reviewed the purpose, methods, and procedures to ensure the protection of participants’ rights and confidentiality. Participants’ informed consent was obtained electronically via online forms during the enrollment process.

Participants were recruited through online notices posted on a dedicated nursing student website and the social media pages of nursing school student councils. The recruitment period was from June 17–27, 2024. The participants were informed of the voluntary nature of their participation, their right to withdraw at any time without consequences, and the strict confidentiality of their data. Survey data were used solely for research purposes, with anonymity ensured. To ensure response quality and prevent duplication, the online survey platform required respondents to answer all questions and did not allow more than one response from the same IP address. The survey was administered via an online link and required approximately 25 minutes to complete.

### Data analysis

The collected data were analyzed using the statistical package PASW 27.0 for Windows (SPSS Inc., Chicago, IL, US). The Shapiro–Wilk test was used to confirm normality, and the Levene test was used to verify homoscedasticity, allowing parametric analyses.

Data from the Likert-type scales used for measuring perceptions, readiness, and performance were treated as continuous interval data for statistical analysis. This approach is consistent with accepted practices in social and health science research because the scales have at least five response options, and the aggregated mean scores calculated from multiple items are considered robust approximations of continuous variables.

The following methods were employed:

1. General characteristics: Frequency, percentage, mean, and standard deviation were used to analyze the participant’s general characteristics.
2. Perceptions and readiness for IPE: Perceptions of the importance of interprofessional CCs and perceptions of performance in interprofessional CCs were analyzed according to participants’ general characteristics using chi-square tests, Fisher’s exact tests, independent *t*-tests, and analysis of variance.
3. Gap analysis: The discrepancy between the perceived importance of and performance in interprofessional CCs was analyzed using paired *t*-tests, the Borich needs assessment, and IPA.
4. Mapping competency quadrants: The LfFM was used to map the CCs into four quadrants based on the X-axis (average required competency level) and the Y-axis (required competency level, encompassing the present average competency level and the discrepancy between perceived importance and actual performance). Subsequently, the study identified the items included in each quadrant.

## Results

### General characteristics of the participants

Participants were predominantly female (86.1%). Regarding satisfaction with their nursing major, 50% were satisfied, while for clinical practice, 47.3% were neutral. The majority of participants (87%) considered IPE necessary, with a strong preference for it to occur during their 3rd–4th years (64.8%). The most desired interaction time was 1–2 hours (47.2%), followed by 3–4 hours (30.6%). The majority of participants (90.7%) had prior IPE experience. Most of these trainings were offered as non-regular, non-credit courses (64.2%), with 55.1% being mandatory. The most common IPE method was joint clinical practice (41.9%), followed by joint lectures (21.4%). Trainings were conducted equally within the same university and in collaboration with other universities. The most frequent collaborators were departments in health-related fields (37.4%), followed by the Department of Medicine (35.2%).

The mean score for the general perception of IPE was 3.83 ± 0.64. In the sub-domains, the score for the perception of competency and autonomy was 3.75 ± 0.73, that for the perceived need for cooperation was 3.96 ± 0.74, that for the perception of actual cooperation was 3.99 ± 0.67, and that for the understanding of others’ values was 3.31 ± 1.20. Overall, participants showed a high level of readiness for IPE, with a mean score of 3.78 ± 0.5. In the sub-domains, teamwork and collaboration scored 4.24 ± 0.6, negative professional identity scored 2.63 ± 1.14, positive professional identity scored 4.06 ± 0.7, and Roles and Responsibilities scored 3.18 ± 0.85.

When evaluating interprofessional CCs, students rated their importance highly across all domains, with a mean score of 4.44 ± 0.51. In the sub-domains, Values and Ethics scored 4.41 ± 0.55, Roles and Responsibilities scored 4.41 ± 0.57, Communication scored 4.50 ± 0.52, and Teams and Teamwork scored 4.4 ± 0.56. Similarly, the perceived performance in interprofessional CCs was also high, with a mean score of 4.25 ± 0.56. In the sub-domains, Values and Ethics scored 4.24 ± 0.52, Roles and Responsibilities scored 4.25 ± 0.64, Communication scored 4.28 ± 0.65, and Teams and Teamwork scored 4.25 ± 0.62 (Table 1).

**Table 1:** General Characteristics and Interprofessional Education-Related Attributes of Participants (*N* = 108).

Characteristics	Categories	*n* (%) or *M* (*SD*)
**Gender**	Male	15 (13.9)
	Female	93 (86.1)
**Satisfaction with a nursing major**	Dissatisfied	8 (7.4)
	Neutral	46 (42.6)
	Satisfied	54 (50)
**Satisfaction with clinical practice**	Dissatisfied	17 (15.7)
	Neutral	51 (47.3)
	Satisfied	40 (37)
**Demand for IPE**	Yes	94 (87.0)
	No	14 (13.0)
**Desired grade of IPE**	1st–2nd	38 (35.2)
	3rd–4th	70 (64.8)
**Desired interaction time between learners during IPE**	None	10 (9.3)
	1–2 hours	51 (47.2)
	3–4 hours	33 (30.6)
	5–6 hours	6 (5.6)
	7–8 hours	3 (2.8)
	≥ 2 days	5 (4.6)
**Experienced IPE**	Yes	98 (90.7)
	No	10 (9.3)
**Credit recognition for IPE**	Regular subject (credit)	37 (35.8)
	Non-regular subject (non-credit)	61 (64.2)
**Autonomy in participating in IPE**	Mandatory	54 (55.1)
	Autonomy	44 (44.9)
**Operating method of IPE**	Joint volunteer activities	6 (6.1)
	Joint workshop/seminar	7 (7.2)
	Joint lecture	21 (21.4)
	Joint clinical practice (case-based learning)	41 (41.9)
	Joint discussion class (problem-based learning, flipped learning)	11 (11.2)
	Joint professional role-play	12 (12.2)
**Affiliation of the participating university**	within the university	49 (50)
	Between different universities	49 (50)
**Departments that participated in IPE**	Department of Medicine	63 (35.2)
	Department of Pharmacy	32 (17.9)
	Dentistry	8 (4.5)
	Oriental medicine	9 (5)
	Health and medically related departments	67 (37.4)
**Perception of IPE**	Total	3.83 ± 0.64
	Competency and autonomy	3.75 ± 0.73
	Perceived need for cooperation	3.96 ± 0.74
	Perception of actual cooperation	3.99 ± 0.67
	Understanding of others’ values	3.31 ± 1.20
**Readiness for IPE**	Total	3.78 ± 0.50
	Teamwork and collaboration	4.24 ± 0.60
	Negative professional identity	2.63 ± 1.14
	Positive professional identity	4.06 ± 0.70
	Roles and responsibilities	3.18 ± 0.85
**Importance of interprofessional CCs**	Total	4.44 ± 0.51
	Values and Ethics	4.41 ± 0.55
	Roles and Responsibilities	4.41 ± 0.57
	Communication	4.50 ± 0.52
	Teams and Teamwork	4.43 ± 0.56
**Performance in interprofessional CCs**	Total	4.25 ± 0.56
	Values and Ethics	4.24 ± 0.52
	Roles and Responsibilities	4.25 ± 0.64
	Communication	4.28 ± 0.65
	Teams and Teamwork	4.25 ± 0.62

CCs = core competencies; IPE = interprofessional education; *M*: mean; *SD*: standard deviation.

### Testing the differences in perceptions of and readiness for Interprofessional Education and in perceived importance of and performance in Interprofessional Education Collaborative Core Competencies according to participants’ general characteristics

The overall perception of IPE (*F* = 10.01, *p* < .001, partial *η*² = 0.16) was significantly higher among participants who reported deep satisfaction with their major (versus those who were neutral or dissatisfied [*F* = 15.16, *p* < .001, partial *η*² = 0.22]) and among those who were deeply satisfied with clinical practice (versus those who were neutral), followed by those who were neutral (versus those who were dissatisfied). The overall perception of IPE (*t* = 2.89, *p* = .005, *d* = 0.62) was significantly higher among par*t*icipants who stated that IPE is necessary (versus those who said it is not). The overall perception of IPE (*t* = 2.31, *p* = .023, *d* = 0.62) was significantly higher among par*t*icipants who participated in IPE mandatorily (versus those who participated autonomously).

Readiness for IPE was notably higher among participants who expressed deep satisfaction with their major (versus those who were neutral or dissatisfied) (*F* = 5.85, *p* = .004, partial *η*² = 0.10) and among those deeply satisfied with their clinical practice (versus those who were dissatisfied) (*F* = 3.62, *p* = .030, partial *η*² = 0.06), among participants who stated that IPE is necessary (versus those who said it is not) (*t* = 3.79, *p* < .001, *d* = 0.56), and among participants who had received IPE (versus those who had not) (*t* = 2.63, *p* = .010, *d* = 0.58). This posi*t*ive correlation between satisfaction and the perceived importance of IPE was a consistent trend across the data. The importance of interprofessional CCs was considerably higher among participants who expressed deep satisfaction with their major (versus those who were neutral or dissatisfied) (*F* = 7.79, *p* < .001, partial *η*² = 0.13), among those deeply satisfied with their clinical practice (versus those who were dissatisfied) (*F* = 4.35, *p* = .015, partial *η*² = 0.08), and among participants who responded that IPE is necessary (versus those who said it is not) (*t* = 2.27, *p* = .025, *d* = 0.50). Additionally, s*t*udents with a higher level of satisfaction and a stronger belief in the necessity of IPE also demonstrated a higher perceived performance. Performance in interprofessional CCs was significantly higher among participants who were deeply satisfied with their major (versus those who were neutral, followed by participants who were neutral versus those dissatisfied) (*F* = 12.10, *p* < .001, partial *η*² = 0.19) and with their clinical practice (versus those who were neutral or dissatisfied) (*F* = 8.61, *p* < .001, partial *η*² = 0.14), and among participants who responded that IPE is necessary (versus those who said it is not) (*t* = 3.09, *p* = .006, *d* = 0.54) (Table 2).

**Table 2:** Differences in Perception of and Readiness for Interprofessional Education and in Perceived Importance of and Performance in Interprofessional Core Competencies Based on Participants’ General Characteristics (*N* = 108).

Characteristics	Categories	Perception of IPE		Readiness for IPE		Importance of interprofessional CCs		Performance in interprofessional CCs	
		*M* ± *SD*	*t* or *F (p)* Cohen’s *d/* *η*²	*M* ± *SD*	*t* or *F(p)* Cohen’s *d/* *η*²	*M* ± *SD*	*t* or *F(p)* Cohen’s *d/* *η*²	*M* ± *SD*	*t* or F*(p)* Cohen’s *d/* *η*²
**Sex**	Male	3.98 ± 0.64	1.00 (.321)	4.01 ± 0.49	1.90 (.060)	4.59 ± 0.52	1.31 (.195)	4.25 ± 0.63	0.02 (.988)
	Female	3.80 ± 0.64	***d* = 0.64**	3.74 ± 0.50	***d* = 0.60**	4.41 ± 0.50	***d* = 0.51**	4.25 ± 0.55	***d* = 0.56**
**Satisfaction with a nursing major**	Dissatisfied	3.35 ± 0.42^a^	10.01 (<.001)	3.43 ± 0.43^a^	5.85 (.004)	3.99 ± 0.70^a^	7.79 (.001)	3.55 ± 0.36^a^	12.10 (<.001)
	Neutral	3.63 ± 0.62^b^	c > a,b (Scheffé)	3.67 ± 0.45^b^	c > a,b (Scheffé)	4.32 ± 0.53^b^	c > a,b (Scheffé)	4.15 ± 0.60^b^	c > b > a (Scheffé)
	Satisfied	4.07 ± 0.58^c^	*η*² = 0.16	3.93 ± 0.51^c^	*η*² = 0.10	4.60 ± 0.39^c^	*η*² = 0.13	4.44 ± 0.44^c^	*η*² = 0.19
**Satisfaction with clinical practice**	Dissatisfied	3.27 ± 0.42^a^	15.16 (<.001)	3.64 ± 0.38^a^	3.62 (.030)	4.25 ± 0.59^a^	4.35 (.015)	3.91 ± 0.57^a^	8.61 (<.001)
	Neutral	3.76 ± 0.56^b^	c > b > a (Scheffé)	3.70 ± 0.47^b^	c > a (Scheffé)	4.36 ± 0.51^b^	c > a (Scheffé)	4.17 ± 0.53^b^	c > a,b (Scheffé)
	Satisfied	4.15 ± 0.62^c^	*η*² = 0.22	3.94 ± 0.55^c^	*η*² = 0.06	4.61 ± 0.41^c^	*η*² = 0.08	4.50 ± 0.48^c^	*η*² = 0.14
**Demand for IPE**	Yes	3.90 ± 0.63	2.89 (.005)	3.85 ± 0.48	3.79 (<.001)	4.48 ± 0.50	2.27 (.025)	4.30 ± 0.56	3.09 (.006)
	No	3.38 ± 0.49	***d*** = 0.62	3.33 ± 0.41	***d*** = 0.56	4.15 ± 0.48	***d*** = 0.50	3.90 ± 0.44	***d*** = 0.54
**Desired year of study for IPE**	1st–2nd year	3.80 ± 0.63	−0.41 (.686)	3.79 ± 0.40	0.13 (.900)	4.47 ± 0.49	0.51 (.611)	4.28 ± 0.55	0.39 (.701)
	3rd–4th year	3.85 ± 0.65	***d*** = 0.64	3.78 ± 0.55	***d*** = 0.61	4.42 ± 0.52	***d*** = 0.51	4.23 ± 0.56	***d*** = 0.56
**Desired interaction time between learners during IPE**	1–2 hours	3.82 ± 0.57	0.71 (.642)	3.73 ± 0.42	1.42 (.213)	4.38 ± 0.48	1.52 (.179)	4.16 ± 0.50	1.64 (.144)
	3–4 hours	3.94 ± 0.65	*η*² = 0.04	3.96 ± 0.55	*η*² = 0.08	4.54 ± 0.43	*η*² = 0.08	4.34 ± 0.53	*η*² = 0.09
	5–6 hours	3.53 ± 0.60		3.43 ± 0.55		4.03 ± 0.72		3.93 ± 0.72	
	7–8 hours	3.76 ± 1.28		3.84 ± 1.04		4.44 ± 0.91		4.17 ± 1.78	
	≥ 2 days	3.84 ± 0.80		3.74 ± 0.40		4.76 ± 0.17		4.73 ± 0.31	
**Experienced IPE**	Yes	3.90 ± 0.64	1.71 (.064)	3.82 ± 0.49	2.63 (.010)	4.44 ± 0.51	0.66 (.508)	4.26 ± 0.56	0.82 (.417)
	No	3.49 ± 0.51	***d*** = 0.63	3.37 ± 0.48	***d*** = 0.58	4.32 ± 0.53	***d*** = 0.51	4.10 ± 0.57	***d*** = 0.56
**Participation in IPE**	Mandatory	3.93 ± 0.65	2.31 (.023)	3.85 ± 0.54	1.55 (.125)	4.48 ± 0.54	1.53 (.130)	4.32 ± 0.57	1.88 (.063)
	Autonomy	3.68 ± 0.57	***d*** = 0.62	3.70 ± 0.43	***d*** = 0.60	4.33 ± 0.46	***d*** = 0.51	4.11 ± 0.53	***d*** = 0.55

*M*: mean; *SD*: standard deviation; *Fisher’s exact test; IPE: interprofessional education; a, b, c: the different lowercase letters within the lines represent the statistical analysis outcomes of the mean differences and scores

### Gap between perceived importance of and performance in interprofessional CCs

The mean score for the importance of interprofessional CCs was 4.44 ± 0.51 out of 5. Among the sub-domains, Communication (4.50 ± 0.52) showed the highest importance, followed by Teams and Teamwork, Values and Ethics, and Roles and Responsibilities. The top five items perceived as the most important by nursing students were as follows: “Clearly articulate one’s roles and duties”; “Practice active listening to encourage other team members to share their ideas and opinions”; “Use constructive feedback to connect, align, and accomplish team goals”; “Utilize communication methods, strategies, and technologies to improve team dynamics, support well-being, and achieve better health outcomes”; and “Contribute to fostering a culture of respect and fairness among one’s colleagues.” This consensus on high importance highlights the perceived necessity of strong foundational collaborative skills.

The mean score for performance in interprofessional CCs was 4.25 ± 0.56 out of 5. The noticeable difference between this score and the importance score suggests a gap between aspiration and practical capability. Sub-domain performance showed a similar pattern, with Communication scoring the highest, followed by Roles and Responsibilities, Teams and Teamwork, and Values and Ethics. The top five items with the highest performance were as follows: “Foster a workplace where interprofessional differences are valued, career satisfaction is encouraged, and well-being is prioritized”; “Maintain expertise in one’s field to effectively contribute to interprofessional care”; “Coordinate team efforts to deliver safe, effective care and achieve the best possible health outcomes”; “Foster a shared understanding of common goals”; and “Use constructive feedback to connect, align, and accomplish team goals.”

The bottom five items demonstrating the lowest performance were as follows: “Promote the values and interests of individuals and groups in the collective efforts of various disciplines and residents’ health planning for healthcare delivery and optimum health”; “Champion social justice and health equity for people of all ages and communities”; “Describe team development and evidence-based processes”; “Value diversity, identity, culture, and differences”; and “Collaborate with sincerity and integrity to advance health equity and improve health outcomes.” Four of these items fell within the Values and Ethics domain, suggesting potential weaknesses in the practical application of social responsibility and ethical principles.

Analysis of the gap between the perceived importance of and performance in interprofessional CCs showed significant differences in 25 out of 33 items. This widespread discrepancy confirms the need for targeted curricular intervention (Table 3). These results indicate that the largest practical deficits are in highly valued communication and professional clarity skills.

**Table 3:** Difference between the Perceived Importance of, and Performance in, Interprofessional Education Collaborative Core Competencies (*N* = 108).

No	Domain/Item	Training requirement	Training experience	Gap	Paired *t* (*p)*	Borich score	Rank
		*M* ± *SD*	*M* ± *SD*	*M* ± *SD*			
Overall		4.44 ± 0.51	4.25 ± 0.56	0.18 ± 0.36	5.29 (<.001)	0.19	
Values and Ethics		4.41 ± 0.55	4.24 ± 0.52	0.17 ± 0.46	3.72 (<.001)	0.17	3
1	Promote shared values in collective care	4.17 ± 0.84	3.97 ± 0.83	0.19 ± 0.92	2.19 (.031)	0.20	12
2	Champion social justice and health equity	4.18 ± 0.73	4.05 ± 0.86	0.13 ± 0.90	1.50 (.136)		
3	Respect dignity, privacy, identity, autonomy, and confidentiality	4.44 ± 0.66	4.23 ± 0.77	0.21 ± 0.84	2.62 (.010)	0.21	9
4	Value diversity, identity, culture, and differences	4.45 ± 0.68	4.17 ± 0.80	0.29 ± 0.91	3.29 (.001)	0.28	4
5	Recognize influence of profession on team dynamics	4.45 ± 0.65	4.29 ± 0.68	0.17 ± 0.68	2.56 (.012)	0.16	18
6	Collaborate with integrity to advance health equity	4.38 ± 0.73	4.18 ± 0.81	0.20 ± 0.90	2.34 (.021)	0.20	12
7	Demonstrate trust, empathy, respect, and compassion	4.44 ± 0.66	4.30 ± 0.69	0.14 ± 0.72	2.02 (.046)	0.14	21
8	Uphold ethical standards in collaborative care	4.43 ± 0.71	4.28 ± 0.66	0.14 ± 0.71	2.05 (.043)	0.15	20
9	Maintain own professional expertise	4.49 ± 0.68	4.35 ± 0.60	0.14 ± 0.68	2.14 (.035)	0.14	21
10	Foster culture of respect and fairness	4.56 ± 0.70	4.42 ± 0.70	0.14 ± 0.80	1.80 (.075)		
11	Foster a workplace that values differences and well-being	4.54 ± 0.60	4.46 ± 0.59	0.07 ± 0.68	1.13 (.260)		
Roles and Responsibilities		4.41 ± 0.57	4.25 ± 0.64	0.16 ± 0.51	3.34 (.001)	0.16	4
12	Integrate team expertise for person-centered care	4.41 ± 0.71	4.22 ± 0.79	0.19 ± 0.78	2.48 (.002)	0.19	14
13	Work collaboratively within and beyond the healthcare system	4.40 ± 0.70	4.30 ± 0.71	0.10 ± 0.59	1.78 (.078)		
14	Incorporate complementary expertise and social determinants of health	4.48 ± 0.63	4.29 ± 0.71	0.19 ± 0.68	2.99 (.003)	0.19	14
15	Clearly distinguish roles and responsibilities of team members	4.39 ± 0.68	4.21 ± 0.85	0.18 ± 0.94	1.95 (.053)		
16	Engage in teamwork with cultural humility	4.40 ± 0.61	4.24 ± 0.73	0.16 ± 0.66	2.49 (.014)	0.16	18
Communication		4.50 ± 0.52	4.28 ± 0.65	0.22 ± 0.53	4.40 (<.001)	0.22	1
17	Clearly articulate own roles and duties	4.59 ± 0.58	4.28 ± 0.73	0.31 ± 0.87	3.75 (<.001)	0.31	3
18	Utilize communication methods/technologies to optimize team function	4.56 ± 0.60	4.23 ± 0.82	0.32 ± 0.77	4.37 (<.001)	0.33	2
19	Communicate genuinely without discipline-specific terminology	4.29 ± 0.85	4.25 ± 0.76	0.04 ± 0.93	0.42 (.679)		
20	Foster a shared understanding of team goals	4.51 ± 0.63	4.31 ± 0.71	0.19 ± 0.69	2.93 (.004)	0.20	12
21	Practice active listening	4.57 ± 0.64	4.30 ± 0.74	0.28 ± 0.68	4.24 (<.001)	0.27	5
22	Give and receive constructive feedback	4.57 ± 0.57	4.31 ± 0.77	0.26 ± 0.73	3.7 (<.001)	0.26	5
23	Reflect on own position, influence, role, expertise, and culture to improve communication	4.41 ± 0.74	4.24 ± 0.78	0.17 ± 0.63	2.73 (.007)	0.17	17
Teams and Teamwork		4.43 ± 0.56	4.25 ± 0.62	0.18 ± 0.38	4.92 (<.001)	0.18	2
24	Understand team development and evidence-based processes	4.47 ± 0.65	4.07 ± 0.89	0.40 ± 0.82	5.05 (<.001)	0.40	1
25	Value varied experiences, expertise, cultures, positions, influence, and roles of team members	4.43 ± 0.61	4.29 ± 0.75	0.14 ± 0.70	2.05 (.043)	0.14	21
26	Engage in collaborative critical thinking, resolving challenges, and decision-making	4.40 ± 0.70	4.27 ± 0.69	0.13 ± 0.64	2.10 (.038)	0.13	25
27	Employ shared leadership strategies	4.36 ± 0.78	4.28 ± 0.81	0.08 ± 0.69	1.26 (.209)		
28	Apply conflict management strategies	4.42 ± 0.70	4.24 ± 0.83	0.18 ± 0.76	2.41 (.018)	0.18	16
29	Reflect on individual and team performance	4.52 ± 0.63	4.30 ± 0.65	0.22 ± 0.59	3.95 (<.001)	0.22	8
30	Share accountability for team outcomes	4.44 ± 0.65	4.30 ± 0.75	0.15 ± 0.62	2.47 (.015)	0.14	21
31	Coordinate care for safe, effective outcomes	4.40 ± 0.68	4.34 ± 0.69	0.06 ± 0.68	0.85 (.399)	0.27	
32	Operate from unified framework for resilience, wellness, safety, and efficiency	4.43 ± 0.64	4.22 ± 0.74	0.20 ± 0.56	3.78 (<.001)	0.21	9
33	Address organizational barriers to team effectiveness	4.43 ± 0.66	4.20 ± 0.72	0.22 ± 0.57	4.06 (<.001)	0.23	7

*M*: mean; *SD*: standard deviation

### Training needs for interprofessional CCs using the Borich needs assessment

A paired *t*-test was conducted to verify the gap between the perceived importance of and performance in interprofessional CCs among nursing students. The analysis revealed a significant difference in 25 out of 33 items. These items were subsequently analyzed using the Borich needs assessment.

The overall gap between perceived importance and performance across all competencies was 0.18 ± 0.36. The analysis showed that the domain in which the students showed the greatest need was Communication, with an average Borich score of 0.23, while Values and Ethics was the one in which they showed the lowest need (0.17). The higher average Borich score for Communication confirms that this domain represents the most urgent training deficit.

The Borich needs assessment highlighted several individual items with particularly high scores, indicating an urgent need for intervention. The item with the highest score in the Borich needs assessment was “Describe team development and evidence-based processes” (Teams and Teamwork, 0.40), followed by “Utilize communication methods, strategies, and technologies to improve team dynamics, support well-being, and achieve better health outcomes,” (C, 0.30), which also showed the largest raw gap between importance and performance (0.32 ± 0.77). This was followed by multiple items with high scores, including “Clearly articulate one’s roles and duties” (C, 0.29), “Differentiate each team member’s role, scope of practice, and responsibility” (RR, 0.29), and “Describe team development and evidence-based processes” (TT, 0.29). Other high-priority items were “Value diversity, identity, culture, and differences” (VE, 0.28) and “Practice active listening to encourage other team members to share their ideas and opinions” (C, 0.27) (Table 3). The top priorities identified by the Borich analysis are rooted in practical team and communication skills, as well as role understanding. These findings suggest that nursing students perceive the greatest educational need in practical skills related to Communication and to Teams and Teamwork, as well as an understanding of diversity and roles. Accordingly, educational interventions should focus heavily on improving the application of these specific competencies.

### Using IPA to identify the priorities of interprofessional CCs

**Fig 1:**
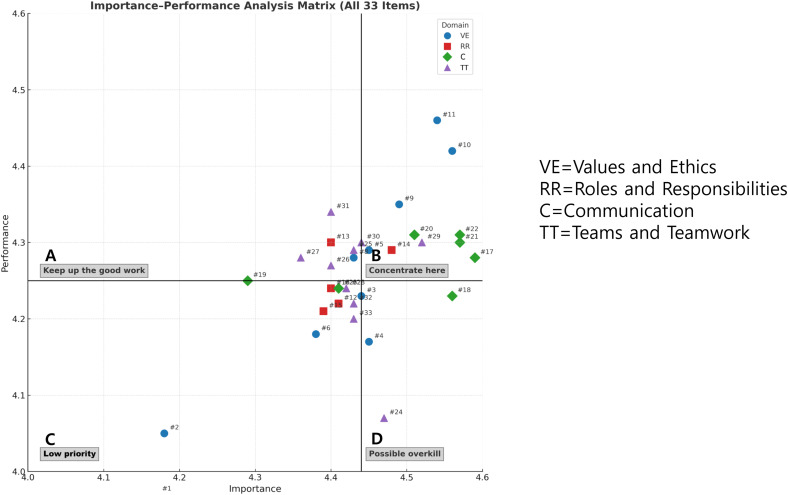
Importance-performance Analysis Matrix of Perceived Importance of and Performance in Interprofessional Education Collaborative Core Competencies VE = Values and Ethics; RR = Roles and Responsibilities; C = Communication; TT = Teams and Teamwork. Note: see Table 3 for details of all items.

An IPA was conducted to classify interprofessional CCs into four quadrants based on their perceived importance and performance. This analysis focused on visualizing areas that nursing students considered highly important but where training experience was insufficient, thereby requiring urgent improvement. Fig 1 illustrates the distribution of CCs into these quadrants. Quadrant A (Concentrate here) contained interprofessional CCs that nursing students considered highly important but showed they needed the most improvement in because of insufficient training. This quadrant contained a total of seven items, which were identified as the most critical areas for educational intervention in this study. These high-priority items were derived from three domains: Roles and Responsibilities (three items), Teams and Teamwork (three items), and Communication (one item).

The majority of the 33 items were concentrated in Quadrant C (Low priority), indicating that most competencies were perceived as below average in both importance and performance.

In contrast, Quadrant D (Possible overkill) had only a few items, which suggests that students are not receiving excessive training in areas they consider to be of low importance, indicating the efficient allocation of educational resources on relevant competencies. In conclusion, the IPA clearly demonstrates that Roles and Responsibilities, Communication, and Teams and Teamwork should be prioritized for curriculum revision to enhance nursing students’ interprofessional collaborative competencies.

Quadrant B (Keep up the good work) contained 19 interprofessional CCs that nursing students both perceived as important and exhibited high performance in (thus requiring continued attention to preserve this high level of achievement). The large number of items in this quadrant indicates success in several areas of current training.

Quadrant C (Low priority) contained interprofessional CCs that nursing students both perceived to be of low importance and demonstrated low performance in (thus requiring no further effort). This quadrant represents areas where minimal curricular attention is needed.

Quadrant D (Possible overkill) contained interprofessional CCs that nursing students did not perceive as important but displayed high performance in, suggesting that their efforts should be redirected to other attributes. However, the small number of items in this quadrant suggests minimal misallocation of training effort.

## Discussion

This study aimed to identify nursing students’ perceptions of and readiness for IPE, as well as their perceived importance of and performance in interprofessional CCs. The findings provide foundational data for designing curricula and educational programs to enhance interprofessional CCs.

The majority of nursing students emphasized the need for IPE, aligning with previous findings that highlight the importance of fostering mutual understanding and collaboration among professionals [29]. However, it should be noted that the self-reporting nature of the survey may be susceptible to social desirability bias. Although no global standard exists for the timing of IPE, phased IPE training tailored to students’ developmental levels is recommended [30]. Prior research also underscores the need for combining diverse methods to develop CCs from beginner to expert levels. Additionally, practice-based IPE within healthcare curricula enhances patient care and safety while fostering positive values [31]. Moreover, highlighting the necessity of selecting IPE topics and expanding opportunities for mutual exchange and communication. Educational goals, purposes, and learning objectives that transcend separate departmental curricula should be reviewed. Participants recognized the role of professional nurses in clinical settings and acquired professional knowledge and skills that lead them to take pride in their professions [32]. Expanding this perspective through systematic IPE curricula could further develop interprofessional CCs.

The analysis of nursing students’ perceived importance of and performance in interprofessional CCs aligns with findings by Park et al. [19], who identified these competencies as being essential for novice nurses, particularly in emergency care. In hospital environments, there are clinical staff members across multiple professions, such as doctors, nurses, paramedics, radiologists, physical therapists, occupational therapists, clinical pathologists, administrative officers, and dietitians [33]. Smooth IPC is directly linked to patient safety and healthcare service quality [34]. Since nurses must have clear communication skills to improve the quality of their work and prevent accidents in clinical settings, these competencies are also stressed as being important in undergraduate curricula [19].

In contrast, the students had the least experience in the Values and Ethics domain in IPE. Although a direct comparison with other groups is limited since the data were not collected from the same group of participants, a previous study analyzed perceptions of interprofessional CCs among pharmacy doctoral students [2]. This highlights a lack of opportunities for training to expand the scope of practice, develop macroscopic perspectives, and address ethical issues [35]. Therefore, there is a clear need for IPE that broadens the scope of care, promotes mutual respect, and establishes ethical guidelines to benefit clinical patients and the broader community.

The use of the LfFM and Borich needs assessment revealed the largest gap in the Teams and Teamwork domain, particularly in evidence-driven approaches to team building and implementation. Anderson et al. [36], who proposed methods for IPE and collaborative practice, emphasized the importance of discussing the team’s model of care to promote awareness of its structures, processes, and roles. This enables effective planning and alignment of methods and direction. To address this, IPE programs should be designed for healthcare students to collaboratively set goals, draw on theoretical models, and develop strategies to achieve these objectives.

A significant gap was also observed in the Communication domain, particularly in the use of strategies, methods, and technologies to strengthen team functionality, well-being, and health outcomes. Medical and nursing students learn the SBAR (situation, background, assessment, recommendation) method during their undergraduate studies to standardize, improve, and promote interprofessional communication. The SBAR is widely recognized for enhancing resilience, increasing job satisfaction, and ensuring patient safety [37].

Additionally, Anderson et al. [36] highlighted the need for adopting shared language among multidisciplinary healthcare professionals to foster collaboration and client-centered outcomes. Their study further noted that exchange between multidisciplinary health professionals—facilitated by shared office spaces or proximity—can enhance understanding of roles and streamline the resolution of client issues.

IPA identified three high-priority items within the Roles and Responsibilities domain that required improvement in Quadrant A (Concentrate here): Work collaboratively with individuals both within and beyond the healthcare system to enhance health outcomes; Clearly distinguish the roles, scopes of practice, and responsibilities of each team member in advancing health outcomes; and Demonstrate cultural humility through effective interprofessional teamwork.

Zenani et al. [38] emphasized that achieving effective IPC requires a shared understanding of the roles across multidisciplinary healthcare disciplines. Rather than adhering to traditional and normatively defined hierarchical structures and roles, the focus should be on the mutual interpretation of roles within social contexts. This involves addressing role-related expectations, execution, and potential conflicts [38,39]. A systematic review by Babin et al. [40], on factors contributing to collaboration between healthcare professionals, highlighted several key systematic organizational factors essential for promoting IPC, including clear shared rules and appropriate information structures, as well as the provision of time, space, and resources for collaborative problem-solving. There was one communication domain item (“Communicate effectively with genuineness and cultural humility, refraining from the use of discipline-specific terminology”) and three Teams and Teamwork domain items (“Engage in collaborative critical thinking, resolving challenges and making decisions as a team”; “Employ shared leadership strategies to enhance team performance and effectiveness”; and “Coordinate team efforts to deliver safe, effective care and achieve the best possible health outcomes”).

To address these priorities, our findings suggest that IPE simulations and clinical practice should be utilized to focus on enhancing communication and teamwork competencies. These programs should provide opportunities to develop mutual communication terms, collaborate on team composition, and coordinate efforts to achieve safety and health goals. Prior research [19] underscores the importance of cultivating communication and teamwork skills through repeated IPE sessions involving clinical cases. Furthermore, the gaps identified by the IPA in the Roles and Responsibilities and Communication domains indicate a need for more practical, hands-on training that cannot be fully addressed through traditional lectures alone. Standardized IPE curricula and educator training programs are necessary to provide systematic opportunities for diverse healthcare undergraduates [30].

This study provides novel evidence by pinpointing specific, high-priority competency gaps using IPA, which can guide future curriculum design and policy formulation highly relevant to South Korea’s nursing education context. The fact that nursing students highly recognize the need for IPE, particularly during their third and fourth years of clinical practice, emphasizes the urgency of developing practice-integrated IPE programs. Crucially, curriculum revision and policy formulation should prioritize the seven areas within Quadrant A of the IPA analysis: Roles and Responsibilities Nos. 13, 15, and 16; Communication No. 19; and Teams and Teamwork Nos. 26, 27, and 31, which require focused, evidence-based attention. First, from a curriculum reform perspective in South Korea, the results advocate for mandated IPE courses. Specifically, based on the IPA priority findings, (1) community-based IPE practice for “Collaborate with others within and outside the health system” (No. 13) should be expanded; (2) workshops and simulations to foster understanding of multidisciplinary roles for “Differentiate each team member#39;s role, scope of practice, and responsibility” (No. 15) should be standardized [31]; (3) IPE modules, including “Practice cultural humility” (No. 16), should be developed to address competencies for interacting with diverse healthcare professionals and multicultural patients [32]; (4) training should be expanded to include practice and feedback on a standardized communication tool like the SBAR [33] and utilize multidisciplinary problem-based learning for “practice team reasoning, problem-solving, and decision-making” (No. 26) and “utilizing shared leadership” (No. 27). From a policy perspective, a strategic and sustained support system must be established to ensure successful implementation, including establishing a dedicated IPE organization at the university headquarters, expanding simulation facilities and equipment, and securing a budget to support multidisciplinary and collaborative faculty IPE training and educational development, all of which are essential [34].

O’Leary et al. [35] suggested the following as key guidelines for the success of practice-based interprofessional education (IPE) for undergraduate students: organizational support, physical resources, reflection of service user needs, educators’ level of practical teaching skills and knowledge, educators’ skills and knowledge for practice-based IPE, securing dedicated training time for IPE, the importance of open communication between multidisciplinary educators, student preparedness, and understanding of interprofessional regulations. As a successful IPE case, King’s College London in the UK significantly improved interprofessional collaboration and trust, as well as patient care competency, by revising the practice-based IPE curriculum, introducing multidisciplinary clinical mentoring, simulation training of patient scenarios with complex problems, and competency-based feedback [36]. At four universities in the UK, simulation-based interprofessional education (Sim-IPE) with medical and nursing students produced significant results in emotional aspects such as students’ professional identity [37]. These practices should be adapted to the specific needs of each country, community, university, and organization to foster nursing talent with collaborative capabilities.

While this study highlights a clear need for IPE, it is important to recognize practical barriers that hinder its implementation in nursing curricula. Specifically, nursing colleges have tight curricula, leaving limited space for introducing new IPE content beyond core nursing theory and practice [38]. Furthermore, selecting training topics that encompass each department#39;s educational objectives is essential [39]. Designing and implementing IPE requires well-trained faculty with experience facilitating interprofessional learning, and yet faculty development and dedicated IPE resources are currently inadequate [40]. Additionally, logistical challenges, such as coordinating schedules across various healthcare disciplines and securing an appropriate interprofessional learning environment, pose significant obstacles [41]. Addressing these barriers is essential to recognizing the need for IPE and effectively implementing it in practice to meet the competencies demanded by modern healthcare [42].

### Limitations and future research

This study has several limitations that should be considered when interpreting the findings. Specifically, its design provided a cross-sectional view of cognition at a single point in time, which prevents assessing changes over time or establishing causal relationships. Furthermore, the use of convenience sampling limits the generalizability of these findings to the broader population of nursing students, while the inclusion of only fourth-year nursing students limits the application of the findings to those in other academic years or healthcare disciplines. Reliance on self-report data also introduces the potential for social desirability bias, as participants may overstate positive perceptions regarding interprofessional collaboration. Additionally, the application of multiple statistical tests in the main analysis may have increased the risk of Type I error (false positives). The modest sample size also limited the statistical power of any subgroup analyses (e.g., stratified by IPE experience), meaning these specific findings should be interpreted with caution. Additionally, the study did not specifically analyze the perceptions of students who considered IPE unimportant in relation to their IPE experience, which would be crucial for understanding these results. It also did not thoroughly explore the specific barriers to IPE integration in nursing education. Variations in university-specific circumstances may limit broader applicability. Future research should, therefore, conduct longitudinal studies to assess changes in perceptions and readiness for IPE over time, establishing causality and tracking the impact of interventions. It should also include students from various healthcare disciplines and different academic years to gain a more comprehensive understanding of IPE training needs, along with analyzing student perceptions based on the specific types of IPE they have experienced, which would be crucial for understanding the results. Future studies should conduct a detailed analysis of students who said that IPE was not important, comparing their responses with those who had never had IPE. This would be a crucial next step to better understand the factors influencing their perceptions. Furthermore, a thorough exploration of the specific barriers to IPE integration within nursing curricula is warranted, as the present study primarily identified areas for improvement. By addressing these recommendations and limitations, future research should assess participant needs and educator competencies to adapt and refine training programs effectively.

## Conclusion

IPC is essential for providing safe, patient-centered healthcare services, and requires effective IPE. Despite the recognized need for IPE among healthcare students and professionals, its integration into regular coursework is limited. This study provides foundational data on the training needs of nursing students for interprofessional CCs. The results highlight the importance of developing specific competencies related to Roles and Responsibilities and collaboration teamwork. Accordingly, educational programs should consider fostering shared leadership based on mutual respect and preparing students to work collaboratively in diverse healthcare settings. This study serves as a foundation for further research and curriculum development efforts aimed at enhancing interprofessional education in nursing.

## Supporting information

S1 Data Raw Data. (XLSX)
